# Blood Pressure Nomograms for Children and Adolescents by Age and Body Mass Index in Tehran, Iran

**Published:** 2017-03

**Authors:** Neamatollah ATAEI, Masoud BAIKPOUR, Mostafa HOSSEINI, Mahmoud YOUSEFIFARD, Mohammad FAYAZ, Fatemeh ATAEI, Arash ABBASI

**Affiliations:** 1. Pediatric Chronic Kidney Disease Research Center, The Children’s Hospital Medical Center, Tehran University of Medical Sciences, Tehran, Iran; 2. Dept. of Pediatric Nephrology, The Children’s Hospital Medical Center, Faculty of Medicine, Tehran University of Medical Sciences, Tehran, Iran; 3. Dept. of Medicine, School of Medicine, Tehran University of Medical Sciences, Tehran, Iran; 4. Dept. of Epidemiology and Biostatistics, School of Public Health, Tehran University of Medical Sciences, Tehran, Iran; 5. Physiology Research Center and Dept. of Physiology, Faculty of Medicine, Iran University of Medical Sciences, Tehran, Iran; 6. Dept. of Nuclear Medicine, Taleghani Hospital, Shahid Beheshti University of Medical Sciences, Tehran, Iran

**Keywords:** Blood pressure, Nomograms, References, Body mass index

## Abstract

**Background:**

Normal standard references of blood pressure (BP) for children and adolescents should be constructed according to anthropometric indices. Therefore*,* we aimed to produce BP reference percentiles by body mass index (BMI).

**Methods:**

Data on demographic characteristics, anthropometric indices and BP values of 16246 3–18-year-old children and adolescents from 3 cross-sectional studies conducted in Tehran were included. To justify the need for BMI adjustment, quantile regression model was applied for different percentiles of systolic and diastolic BPs with age, sex, and the corresponding BMI percentiles. Then, Age- and sex-specific BP nomograms were constructed according to BMI.

**Results:**

All regression coefficients for BMI percentiles were significant in quantile regression of BPs, confirming the necessity for BMI-adjusted nomograms of BP. The BP percentiles for each gender by age and BMI are presented. All the BP percentiles rose steadily in all BMI percentiles with minor discrepancies between the two genders. As observed, the prevalence of hypertension is estimated to be lower among the lean subjects and higher among overweighs when the BMI-adjusted BP curves are considered.

**Conclusion:**

The reference database constructed in this survey is the first Iranian BP reference by age and BMI in children and adolescents, from it concluded that BMI-adjusted BP curves depict a more precise picture of the hypertension prevalence and present a more reliable classification standard for hypertension.

## Introduction

Hypertension as a major cause of disability and premature deaths all around the world (13.5% of the premature deaths and 6% of the total global DALYs) is one of the most common risk factors for cardiovascular diseases and an important part of the worldwide burden of disease is attributable to high blood pressure (BP) (1). Hereon, major attention has been drawn to hypertension in adults and children and it has become a priority for the health policy makers to manage. Since early detection of hypertension is of utmost importance to help reduce its various complications, blood pressure assessment is now considered as an essential part of routine physical examination (2).

Cardiovascular accidents most frequently happen after the age of fifty but evidence on pathophysiologic and epidemiologic aspects of the disease is available suggesting that hypertension and risk factors for cardiovascular disease originate in childhood (3). Some studies have also found a strong correlation between increased blood pressure levels in childhood and hypertension in adulthood (4–6). Considering the undeniable importance of primary prevention, many researchers have shown interest in evaluation of blood pressure trends in childhood and adolescence.

Moreover, BP variations have been observed among different ethnicities and races (7–10), therefore, standard nomograms derived from a specific population might not be suitable for others and local reference data should be considered instead (9–14). In this regard, the United States’ Task Force on BP Control in Children presented an extended series on age- and height-related BP values from birth to 18 yr to establish reference data for BP in children and adolescents (15–17). Many other countries have also presented similar reference data (7, 8, 10, 18, 19). Although few studies have presented references for systolic and diastolic BP measurements in Iranian population of children and adolescents (13, 20–22), only one study, was nationally representative and included the entire age groups of children and adolescents (23). In their survey they only presented the BP percentiles by age and height but since many studies have confirmed the stronger relation of BP with BMI (24–26), we aimed to construct BP percentiles by this factor for children and adolescents. To the extent of our knowledge, this is the first study looking at the BMI-related age-specific BP reference values in children and adolescents worldwide.

## Materials and Methods

### Study Population

Our study population included 16246 children and adolescents from Tehran, which as the capital city of Iran, was proven to have a nationally representative population for the entire country (27). Subjects from three cross-sectional studies were included to constitute our study population of children and adolescents aged 3 to 18 yr old. All the three studies were conducted on randomized samples from 20 different geographical areas of Tehran. The first survey was conducted during Nov 2000 to Nov 2002 and included 8848 7 to 12-year-old primary school children from Tehran. The second one, conducted in 2004, included 6017, 12 to 18-year-old children and adolescents from secondary and high schools of Tehran. In the last survey, conducted in 2010, a 2-stage cluster sampling method was used to select 2107 subjects including 1 month to 2-year-old infants from health centers and 2 to 7-year-old children from kindergartens (28).

Being a healthy child, as the inclusion criterion was defined as 1) having a normal general appearance; 2) no documented underlying disease; 3) no history of cardiovascular problems; 4) no history of antihypertensive drugs. Trained interns of the medical center were responsible for data collection. Informed consent was obtained from the parents or guardians of the subjects.

Our sampling methods are further explained in our previous publications (13, 28, 29).

### Blood pressure measurements

A standard mercury sphygmomanometer (Model 1002/ Presameter, Riester, Germany) was used to measure BPs of all the children in a wakeful state after at least a 5-min rest, in a comfortable sitting position.

The proper cuff was selected with a bladder long enough to cover 80%–100% of the arm circumference and width of approximately 40% the arm length. The right arm was positioned at heart level with the cuff placed around the arm leaving the antecubital fossa free for auscultation. While checking the radial pulse, the bladder was inflated to a level that occludes the artery and stops the pulse. With the stethoscope placed over the brachial artery in the antecubital fossa, the cuff was deflated. The pressure at which, the first Korotkoff (K1) sound was heard, was recorded as the systolic BP. For children under 12 yr old, the pressure at the onset of the K4 sound, and for adolescents aged 13 to 18 yr the pressure at the onset of the K5 sound was considered as the standards for diastolic BPs. BP was measured twice for each subject with an interval of 30 sec and the mean of the two values was recorded for data analysis.

### Height and weight measurements

Children aged 3 to 6 yr old were weighed using a SECA scale (USA, model 760) with an accuracy of 500 gr and their standing height with an accuracy of 1 mm was measured by a SECA mechanical measuring tape (USA, model 206).

For school-aged children height was measured with the student standing upright, barefoot, with the heels and back against a vertical SECA stadiometer (Germany, model 207). Weight without shoes and heavy outer clothing was measured via a daily-calibrated SECA balanced scale (Germany, model 710).

### Body Mass Index (BMI)

Body mass (kg) divided by the square of the subject’s height (m) was recorded as BMI.

### Age-sex-specific percentile values

In order to evaluate the relationships between BMI with blood pressure measurements and age, first the age-sex-specific normal deviations (Z_α_) of BMI were calculated (30). Then, the age-sex-specific percentile values of BMI were derived through mounting the computed Z_α_ in the standard normal distribution and calculating the corresponding percentile value.

### Construction of the BP nomograms according to age, sex and BMI

Two separate models were constructed for SBP and DBP of each gender to present BP percentiles as a function of age and BMI. At first, the latent moderated structural (LMS) equations method was applied to model BMI percentiles with age for males and females (31). Then the reference curves for children and adolescents were fitted by age and BMI simultaneously. The Generalized Additive Models for Location Scale and Shape (GAMLSS) with the Box-Cox-Cole-Green distribution family (32–34) were fitted with GAMLSS 4.2-0 in the free statistical software R 2.15.2 (http://www.R-project.org) (23).

Finally, we compared the fitted percentile curves with the reference values of USA (15), Germany (19), Turkey (10), Great Britain (8), China (18) and Saudi Arabia (7).

## Results

Blood pressures, height, and weight of 8381 boys and 7865 girls aged 3–18 yr were measured. Table 1 demonstrates the baseline characteristics of the study population. As can be seen, the means of weight, height, and BMI in all the age groups were higher among boys compared to girls. As mentioned, BP was measured twice for each subject. The differences between the two measurements were insignificant. The means of absolute differences for boys and girls ranged 0.22–1.48 mmHg and 0.3–1.58 mmHg, respectively. Therefore, the mean of the two measurements was computed and used for the analysis. The mean of diastolic and systolic BPs is almost higher among boys for all the age groups, except for the SBP in age group of 7–12 yr where girls have a slightly higher mean.

**Table 1:** Baseline characteristics of the reference population of children and adolescents (8381 boys and 7865 girls)

**Characteristics**	**Age (yr)**		
	**3–6**	**7–12**	**13–18**
**Children included, *n***			
Boys	746	4505	3130
Girls	635	4698	2532
**Weight, mean (SD), kg**			
Boys	16.96 (4.06)	29.50 (8.38)	58.33 (15.15)
Girls	15.86 (3.50)	27.73 (7.92)	48.28 (11.51)
**Height, mean (SD), cm**			
Boys	103.3 (9.5)	133.2 (10.3)	165.6 (11.5)
Girls	102.1 (8.8)	131.0 (10.3)	155.3 (8.7)
**BMI, mean (SD), kg/m^2^**			
Boys	15.73 (2.07)	16.36 (2.89)	21.05 (4.10)
Girls	15.12 (2.07)	15.89 (2.85)	19.82 (3.70)
**SBP, mean (SD), mmHg**			
Boys	93.56 (10.24) (10.61)	107.24 (8.98)	115.35 (11.51) (11.72)
Girls	91.50 (8.79) (10.61)	107.45 (8.90)	108.97 (10.66) (11.72)
**DBP, mean (SD), mmHg**			
Boys	55.35 (9.61) (10.61)	63.69 (9.49) (10.61)	72.41 (7.87) (11.72)
Girls	54.41 (8.48) (10.61)	63.34 (9.57) (10.61)	71.21 (9.11) (11.72)
**Overweight, n (%)**			
Boys	119 (15.9)	436 (9.7)	572 (18.3)
Girls	70 (11.0)	325 (7.9)	288 (11.5)

To justify the need for BMI adjustment, quantile regression model was performed for different percentiles of systolic and diastolic blood pressures with age, sex, and corresponding BMI percentile values. As illustrated in Table 2, all coefficients for BMI percentiles are statistically significant at *P*<0.001, confirming the necessity for BMI-adjusted nomograms of blood pressure.

**Table 2:** Estimated quantile regression for blood pressure measurements according to age, 85^th^ percentile of body mass index (BMI) and gender

**Parameters**	**25^th^**		**50^th^**		**75^th^ / 85^th^**		**95^h^**	
	**Coeff (±SE)**	***P***	**Coeff (±SE)**	***P***	**Coeff (±SE)**	***P***	**Coeff (±SE)**	***P***
**Systolic Blood Pressure**								
Age (yr)	1.38 (0.03)	<0.001	1.46 (0.02)	<0.001	1.80 (0.02)	<0.001	1.92 (0.04)	<0.001
Sex	−1.61 (0.21)	0.495	−1.70 (0.19)	<0.001	−0.97 (0.15)	<0.001	−0.15 (0.28)	0.84
BMI (Percentile)	0.06 (0.003)	<0.001	0.05 (0.003)	<0.001	0.04 (0.002)	<0.001	0.07 (0.005)	<0.001
Intercept	84.8 (0.41)	<0.001	91.2 (0.33)	<0.001	95.3 (0.23)	<0.001	96.8 (0.44)	<0.001
R^2^	0.1365		0.1569		0.2204		0.2291	
**Diastolic Blood Pressure**								
Age (yr)	1.53 (0.03)	<0.001	1.45 (0.02)	<0.001	1.52 (0.02)	<0.001	1.54(0.02)	<0.001
Sex	−0.09 (0.20)	0.657	−1.06 (0.18)	0.554	0.60 (0.12)	<0.001	1.16 (0.16)	<0.001
BMI (Percentile)	0.05 (0.003)	<0.001	0.04 (0.003)	<0.001	0.03 (0.002)	<0.001	0.04 (0.003)	<0.001
Intercept	41.3 (0.35)	<0.001	49.0 (0.31)	<0.001	55.3 (0.21)	<0.001	57.6 (0.31)	<0.001
R^2^	0.1467		0.1726		0.2622		0.2529	

All coefficients (Coeff) for BMI percentiles are statistically significant at *P*<0.001.

BP percentiles for boys and girls according to age and BMI are presented in Tables 3 and 4. No additional tables are required to interpret the presented information since the 5^th^, 10^th^, 25^th^, 50^th^, 85^th^, 90^th^ and 95^th^ percentiles of BMI are given in kg/m^2^.

**Table 3:** Blood pressure values for boys according to age and body mass index (BMI)

		**SBP (mm Hg)**				**DBP (mm Hg)**			
**Age (Year)**	**BMI (**kg/m^2^**)**	**50^th^ Percentile (Median)**	**90^th^ Percentile**	**95^th^ Percentile**	**99^th^ Percentile**	**50^th^ Percentile (Median)**	**90^th^ Percentile**	**95^th^ Percentile**	**99^th^ Percentile**
3	13.5	86	95	97	101	51	60	62	67
3	14	86	95	97	102	51	60	63	67
3	14.8	87	96	98	103	51	61	63	68
3	16	87	97	99	104	52	62	64	68
3	18.4	89	98	101	106	53	63	66	70
3	18.9	89	99	101	106	53	63	66	70
3	19.9	90	100	102	107	54	64	67	71
4	13.1	90	99	102	106	53	62	65	69
4	13.6	90	99	102	107	53	63	65	69
4	14.5	91	100	103	107	53	63	66	70
4	15.6	91	101	103	108	54	64	66	71
4	18	93	103	105	110	55	65	68	72
4	18.5	93	103	106	110	55	66	68	73
4	19.6	94	104	106	111	56	66	69	73
5	12.8	93	103	106	110	55	64	67	71
5	13.3	94	103	106	111	55	65	67	71
5	14.2	94	104	107	111	56	65	68	72
5	15.3	95	105	107	112	56	66	68	73
5	17.8	97	107	109	114	57	67	70	74
5	18.3	97	107	110	114	57	68	70	74
5	19.4	97	108	110	115	58	68	71	75
6	12.6	97	107	109	114	57	66	69	73
6	13.1	97	107	110	114	57	67	69	73
6	14	97	107	110	115	57	67	70	74
6	15.1	98	108	111	116	58	68	70	75
6	17.7	100	110	113	118	59	69	72	76
6	18.1	100	110	113	118	59	69	72	76
6	19.3	101	111	114	119	60	70	72	77
7	12.4	99	110	112	117	59	68	70	75
7	12.9	100	110	113	118	59	68	71	75
7	13.8	100	111	113	118	59	69	71	75
7	15	101	111	114	119	60	69	72	76
7	17.7	103	113	116	121	61	71	73	78
7	18.1	103	113	116	121	61	71	73	78
7	19.3	103	114	117	122	62	71	74	78
8	12.4	102	112	115	120	60	70	72	76
8	12.9	102	113	115	121	61	70	72	77
8	13.8	103	113	116	121	61	70	73	77
8	15.1	103	114	117	122	61	71	73	78
8	17.8	105	116	119	124	63	72	75	79
8	18.3	105	116	119	124	63	72	75	79
8	19.5	106	117	120	125	63	73	76	80
9	12.6	104	115	117	123	62	71	74	78
9	13.1	104	115	118	123	62	71	74	78
9	14	105	116	119	124	63	72	74	78
9	15.3	106	116	119	125	63	73	75	79
9	18.2	107	118	121	127	64	74	76	81
9	18.8	108	119	122	127	64	74	76	81
9	20.1	108	119	122	128	65	75	77	81
10	12.8	106	117	119	125	64	73	75	79
10	13.4	106	117	120	125	64	73	75	79
10	14.4	107	118	120	126	64	73	76	80
10	15.8	107	118	121	127	65	74	76	81
10	18.9	109	120	123	129	66	75	78	82
10	19.5	110	121	124	129	66	76	78	82
10	20.9	110	121	125	130	67	76	79	83
11	13.2	107	118	121	126	65	74	76	80
11	13.8	107	118	121	127	65	74	77	81
11	14.9	108	119	122	128	66	75	77	81
11	16.4	109	120	123	129	66	75	78	82
11	19.8	111	122	125	131	67	77	79	83
11	20.4	111	122	126	131	68	77	79	84
11	22	112	123	126	132	68	78	80	84
12	13.7	108	119	122	128	67	75	78	82
12	14.4	109	120	123	128	67	76	78	82
12	15.5	109	121	124	129	67	76	78	82
12	17.2	110	122	125	130	68	77	79	83
12	20.9	112	124	127	132	69	78	81	85
12	21.6	113	124	127	133	69	78	81	85
12	23.4	114	125	128	134	70	79	81	86
13	14.3	110	121	124	129	68	77	79	83
13	15	110	121	124	129	68	77	79	83
13	16.3	111	122	125	130	69	77	80	84
13	18	112	123	126	132	69	78	80	84
13	22.1	114	126	129	134	71	80	82	86
13	22.9	114	126	129	135	71	80	82	86
13	24.9	115	127	130	136	71	81	83	87
14	14.9	111	122	125	130	69	78	80	84
14	15.6	111	122	125	131	70	78	80	84
14	17	112	123	126	132	70	79	81	85
14	18.9	113	125	128	133	71	79	82	86
14	23.3	116	127	131	136	72	81	83	87
14	24.1	116	128	131	137	72	81	84	88
14	26.3	117	129	132	138	73	82	84	88
15	15.5	112	123	126	132	71	79	81	85
15	16.2	113	124	127	133	71	79	81	85
15	17.7	114	125	128	134	72	80	82	86
15	19.7	115	126	130	135	72	81	83	87
15	24.3	118	129	133	138	74	82	84	88
15	25.3	118	130	133	139	74	83	85	89
15	27.6	119	131	135	141	75	83	86	90
16	15.9	114	125	128	133	72	80	82	86
16	16.7	114	125	129	134	72	80	82	86
16	18.2	115	127	130	135	73	81	83	87
16	20.3	117	128	131	137	73	82	84	88
16	25.2	120	132	135	141	75	83	86	89
16	26.3	120	132	135	141	75	84	86	90
16	28.8	122	134	137	143	76	84	87	91
17	16.3	115	126	129	135	73	81	83	87
17	17.1	115	127	130	136	73	81	83	87
17	18.7	116	128	131	137	74	82	84	88
17	20.9	118	130	133	139	75	83	85	88
17	26	121	133	137	143	76	84	86	90
17	27.1	122	134	138	143	76	85	87	91
17	29.7	124	136	139	145	77	85	88	91
18	16.6	116	127	130	136	74	82	84	87
18	17.5	116	128	131	137	74	82	84	88
18	19.1	118	129	133	138	75	83	85	88
18	21.4	119	131	134	140	76	83	85	89
18	26.7	123	135	139	145	77	85	87	91
18	27.9	124	136	140	146	78	86	88	91
18	30.6	126	138	141	148	78	86	88	92

BMI (kg/m^2^) for each age represents the 5th, 10th, 25th, 50th, 85th, 90th, and 95th percentile.

**Table 4:** Blood pressure values for girls according to age and body mass index (BMI)

		**SBP (mm Hg)**				**DBP (mm Hg)**			
**Age (Year)**	**BMI (kg/m^2^)**	**50^th^ Percentile (Median)**	**90^th^ Percentile**	**95^th^ Percentile**	**99^th^ Percentile**	**50^th^ Percentile (Median)**	**90^th^ Percentile**	**95^th^ Percentile**	**99^th^ Percentile**
3	12.9	85	93	95	99	51	59	62	66
3	13.4	85	93	95	99	51	60	62	66
3	14.4	85	94	96	100	51	60	62	66
3	15.5	86	94	97	101	52	61	63	67
3	17.9	88	96	98	103	53	62	64	68
3	18.3	88	96	99	103	53	62	64	69
3	19.3	89	97	99	104	53	63	65	69
4	12.5	89	98	100	104	52	61	64	68
4	13	89	98	100	105	53	62	64	68
4	14	90	98	101	105	53	62	64	69
4	15.2	90	99	101	106	54	63	65	69
4	17.6	92	101	103	108	55	64	66	70
4	18.1	92	101	103	108	55	64	66	71
4	19.1	93	102	104	109	55	65	67	71
5	12.2	93	102	104	109	54	63	66	70
5	12.7	93	102	105	109	55	64	66	70
5	13.7	94	103	105	110	55	64	66	71
5	14.9	94	103	106	111	55	65	67	71
5	17.5	95	105	107	112	56	66	68	72
5	17.9	96	105	108	112	57	66	68	73
5	18.9	96	106	108	113	57	67	69	73
6	12	96	106	109	113	56	65	68	72
6	12.5	97	106	109	114	56	66	68	72
6	13.4	97	107	109	114	57	66	68	72
6	14.7	98	107	110	115	57	67	69	73
6	17.4	99	109	111	116	58	68	70	74
6	17.8	99	109	112	117	58	68	70	75
6	18.9	100	110	112	117	59	69	71	75
7	11.8	100	109	112	117	58	67	70	74
7	12.4	100	110	112	117	58	67	70	74
7	13.3	100	110	113	118	58	68	70	74
7	14.6	101	111	113	119	59	68	71	75
7	17.4	102	112	115	120	60	70	72	76
7	17.8	102	112	115	120	60	70	72	77
7	19	103	113	116	121	61	70	73	77
8	11.8	102	113	115	121	60	69	71	76
8	12.4	102	113	116	121	60	69	72	76
8	13.4	103	113	116	121	60	70	72	76
8	14.7	103	114	117	122	61	70	73	77
8	17.6	105	115	118	123	62	72	74	78
8	18.1	105	115	118	124	62	72	74	79
8	19.4	106	116	119	125	63	72	75	79
9	12	104	115	118	123	61	71	73	78
9	12.5	105	115	118	123	62	71	74	78
9	13.6	105	116	119	124	62	72	74	78
9	15	106	116	119	125	63	72	75	79
9	18.1	107	118	121	126	64	74	76	81
9	18.6	107	118	121	127	64	74	76	81
9	20	108	119	122	127	65	74	77	81
10	12.3	106	117	120	125	63	73	75	79
10	12.9	106	117	120	126	63	73	75	80
10	14	107	118	121	126	64	73	76	80
10	15.4	107	118	121	127	64	74	77	81
10	18.8	109	120	123	129	66	76	78	83
10	19.3	109	120	123	129	66	76	78	83
10	20.8	110	121	124	130	66	76	79	84
11	12.7	107	118	121	127	65	75	77	81
11	13.3	108	119	122	127	65	75	77	82
11	14.5	108	119	122	128	66	75	78	82
11	16.1	109	120	123	128	66	76	78	83
11	19.6	110	122	125	131	68	77	80	84
11	20.3	111	122	125	131	68	78	80	85
11	21.9	112	123	126	132	68	78	81	86
12	13.3	108	119	122	128	67	76	79	83
12	14	108	119	123	128	67	77	79	83
12	15.2	109	120	123	129	67	77	80	84
12	16.8	110	121	124	130	68	78	80	85
12	20.7	112	123	126	132	69	79	82	86
12	21.3	112	124	127	133	70	80	82	87
12	23.1	113	125	128	134	70	80	83	88
13	14	108	120	123	129	68	78	80	85
13	14.7	109	120	123	129	69	78	81	85
13	16	109	121	124	130	69	79	81	86
13	17.7	110	122	125	131	70	80	82	86
13	21.8	113	124	128	133	71	81	84	88
13	22.5	113	125	128	134	72	82	84	89
13	24.4	114	126	130	136	72	82	85	89
14	14.7	109	120	123	129	70	79	82	86
14	15.4	109	120	124	129	70	80	82	87
14	16.7	110	121	124	130	71	80	83	87
14	18.6	111	122	126	131	71	81	84	88
14	22.8	113	125	129	135	73	83	85	90
14	23.6	114	126	129	135	73	83	86	90
14	25.5	115	127	131	137	74	84	87	91
15	15.3	109	120	123	129	71	81	83	88
15	16	109	121	124	130	72	81	84	88
15	17.5	110	122	125	131	72	82	84	89
15	19.4	111	123	126	132	73	82	85	89
15	23.7	114	126	129	136	74	84	87	91
15	24.5	115	127	130	136	75	84	87	92
15	26.5	116	129	132	138	75	85	88	92
16	15.8	109	120	124	129	72	82	84	89
16	16.6	109	121	124	130	73	82	85	89
16	18	110	122	125	131	73	83	85	90
16	20	111	123	126	132	74	83	86	90
16	24.4	115	127	130	136	75	85	88	92
16	25.1	115	127	131	137	76	86	88	93
16	27.1	117	129	133	139	76	86	89	94
17	16.3	108	120	123	129	73	83	85	90
17	17.1	109	121	124	130	74	83	86	90
17	18.5	110	122	125	131	74	84	86	90
17	20.5	111	123	126	133	75	84	87	91
17	24.9	115	127	130	137	76	86	89	93
17	25.6	115	128	131	137	77	86	89	93
17	27.6	117	130	133	140	77	87	90	94
18	16.7	108	120	123	129	74	84	86	90
18	17.5	108	120	123	129	75	84	86	91
18	18.9	109	121	125	130	75	85	87	91
18	20.9	111	123	126	132	76	85	88	92
18	25.3	115	127	131	137	77	87	89	94
18	26	115	128	131	138	78	87	90	94
18	27.9	117	130	133	140	78	88	91	95

BMI (kg/m^2^) for each age represents the 5th, 10th, 25th, 50th, 85th, 90th, and 95th percentile.

As can be seen, the 85^th^ percentile of BMI is given and the corresponding BPs are modeled because the 85^th^ to 95^th^ percentiles of BMI are defined as overweight. SBP values were mostly higher among the boys except for the ages of 9 through 13 where the curves nearly overlap. The DBP curves are somewhat different. The values of DBP before the age of 8 are higher among boys, but at this age, the curves cross and the DBP values for girls remain higher through the age of 18. Overall, the differences are milder than the SBP curves.

One of the benefits of constructing standard curves of BP by anthropometric indices is that the prevalence of hypertension can be better elucidated. Therefore, next in this article, we focused our assessments on estimating the prevalence of hypertension according to BMI since it is a better indicator of the subjects’ nutritional status and it includes the effects of weight and height as well. The prevalence of hypertension using the US references presented in the fourth task force report for subjects whose BMI were in the <25th, 25th–85th, and >85th percentiles were 7.25, 7.72, and 8.10% for girls and 9.15, 5.24, and 6.92% for boys, respectively. However, when the BMI-adjusted BP percentiles were used, the prevalence of hypertension for the aforementioned BMI percentiles was 3.89, 7.46, and 13.33% for girls and 3.29, 3.93, and 10.03% for boys, correspondingly. As can be seen, when the BMI-adjusted standard curves of BPs are used, the prevalence of hypertension is estimated to be lower among the lean subjects and higher among the over weights. Therefore, adjustment of these curves according to BMI decreases the false positive cases in lean subjects and false negative cases among overweight children and adolescents.

## Discussion

The high prevalence of hypertension and its various complications associated with high morbidity and mortality rates, make it a major public health problem all around the globe (29). Since childhood BP levels are predictive of BP levels in adulthood, was provided normal standard references of BP for children and adolescents according to age, sex, and anthropometric indices (16, 18, 35). The American Academy of Pediatrics Task Force on Blood Pressure published a series of reports on BP levels according to age and height from birth to 18 yr to establish standard references for pediatric BP (15, 36, 37). Advanced statistical methods was applied on the data gathered through the German Health Interview and Examination Survey for Children (38) and Adolescents (KiGGS 2003–2006) (39) and presented standard BP references for non-overweight, 3 to 17-year-old children and adolescents of Germany.

The reference database constructed in this survey is the first BP reference by age and BMI in children and adolescents worldwide. In other studies, the standard curves of BP were only presented by age and gender. For example 5599 Turkish children from birth to 18 yr of age were evaluated (10). These researchers drew normal BP curves for Turkish pediatric population based on the collected data. BP centiles derived were presented from data gathered through examination of 22901 children aged 4 to 23 yr old from Great Britain (8). A similar survey was conducted in their country, Saudi Arabia (7). They constructed BP standard reference percentiles from data gathered from 16226 infants, children, and adolescents from birth to 18 yr of age. From China, (18) a study based on eleven large-scale were conducted cross-sectional BP surveys in their country included 112227 children and adolescents aged 3 to 18 yr old.

**Fig. 1:**
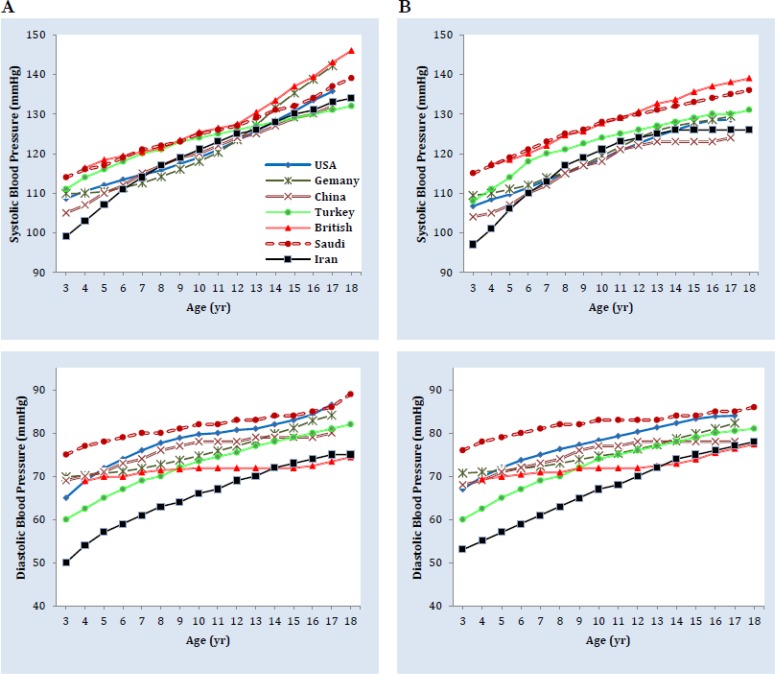
Comparison of the 95^th^ percentile of blood pressure values in boys (A) and girls (B) from several countries. Blood pressure values of Iranian, American (USA), and German children and adolescents correspond to the 50^th^ percentile of body mass index.

We derived the analogous data on children and adolescents aged 3 to 18 from these surveys. For the information to be comparable with each other, only data on the 50^th^ percentile of height from the German references and the 50^th^ percentile of BMI from ours were included. Fig. 1 depicts the 95^th^ percentile of the BP values by age, for all the 7 surveys conducted on this matter. The SBP rose progressively with age in both genders, with the rise being steeper among boys after the age of 13. The DBP curves show slight differences between the two genders.

Great Britain and Saudi Arabia present the highest SBP levels for both genders compared to other countries with minor differences compared to each other until the age of 13, where their curves start to diverge and the Great Britain stays on top. Up to the age of six, Iran has the lowest BP levels among these countries for both genders. From that point on, although our BP levels are not the lowest of all but they are among the 2 or 3 lowest curves presented.

As for the DBP levels, Saudi Arabia, has the highest levels in almost all the ages for both genders. Iranian DBP curve presents the lowest DBP values with an extended difference compared to other countries. Great Britain’s DBP levels present with a mostly horizontal curve downgrading among other curves until it becomes the lowest after the age of 13 yr old.

Furthermore, we evaluated the prevalence of hypertension based on different references and among various groups of the study population including boys and girls in each of the <25^th^, 25^th^–85^th^, and >85^th^ percentiles of BMI. The overall prevalence among girls based on US references (7.66%) was slightly higher than the prevalence estimated based on Iranian BMI-adjusted references (7.38%). Both figures among boys were lower than girls, however, the disagreement between the estimates was greater among boys compared to girls with 6.35% based on US references and 4.70% based on Iranian BMI-adjusted references.

When the standard curves of BP were constructed according to BMI, the prevalence of hypertension increased among the overweight subjects and decreased in the lean population, preventing misinterpretations of the BP measurements.

Major strengths of our survey include the large and nationally representative sample, covering a wide range of ages, standardized measurements of BP, weight, and height, application of a BP measuring device validated in children, measuring BPs twice for each subject and the modeling by age and BMI simultaneously with advanced statistical methods.

Measurements having been performed by human, imposed end-digit preference on our data, i.e. some values happened to be more frequently reported such as those ending in 0 or 5 (40). It does not meet the goodness of fit criteria and changes the fitted models along with the prevalence of hypertension calculated upon these fitted curves (41).

## Conclusion

The reference curves constructed in this study is the first Iranian BP reference by age, and BMI, covering children and adolescents aged 3 to 18 yr old. BMI-adjusted BP curves depict a precise picture of the hypertension prevalence in children and adolescents and present a reliable meticulous classification standard for hypertension.

## Ethical considerations

Ethical issues (Including plagiarism, informed consent, misconduct, data fabrication and/or falsification, double publication and/or submission, redundancy, etc.) have been completely observed by the authors.
